# Diacylglycerol Kinase ε in Adipose Tissues: A Crosstalk Between Signal Transduction and Energy Metabolism

**DOI:** 10.3389/fphys.2022.815085

**Published:** 2022-01-27

**Authors:** Tomoyuki Nakano, Kaoru Goto

**Affiliations:** Department of Anatomy and Cell Biology, School of Medicine, Yamagata University, Yamagata, Japan

**Keywords:** adipose tissue, adipose triglyceride lipase, beige adipogenesis, diacylglycerol kinase, glucose tolerance, obesity, uncoupling protein 1

## Abstract

Diacylglycerol (DG) is unique in lipid metabolism because it serves not only as an intermediate product for triglyceride synthesis, but also as a signaling molecule that activates proteins containing DG-responsive elements, such as protein kinase C. Consequently, DG acts as a hub between energy metabolism and intracellular signaling. Of DG metabolizing pathways, DG kinase (DGK) phosphorylates DG to produce phosphatidic acid, which also serves as a second messenger. Several lines of evidence suggest that DGK is deeply involved in metabolic diseases such as obesity and insulin resistance. Of DGK isozymes, DGKε is simplest in terms of structure, but it is characterized by substrate specificity toward arachidonoyl-DG. Recently, we have reported that DGKε deficiency promotes adipose tissue remodeling in mice during the course of high fat diet (HFD) feeding regimen including obesity, insulin resistance, and beige adipogenesis. DGKε ablation engenders altered expression of other lipid metabolizing enzymes, including adipose triglyceride lipase (ATGL), hormone-sensitive lipase (HSL), and diacylglycerol acyltransferase (DGAT). Subcellular localization of DGKε in the endoplasmic reticulum suggests involvement of this isozyme in lipid energy homeostasis. This review presents current findings of DGKε in lipid-orchestrated pathophysiology, especially unique phenotypes of DGKε-knockout mice in the early and late stages of obesogenic conditions.

## Introduction

Lipid is an indispensable constituent of cells. It composes biological membranes surrounding the cell itself, the nucleus, and subcellular organelles. In addition, lipid is stored as an energy source in a specialized organelle called lipid droplet. Of lipids, diacylglycerol (DG), a basic structure of phospholipids, comprises at least 50 molecular species (Sakane et al., 2018) containing two acyl chains of various combinations at sn-1,2, sn-1,3, or sn-2,3 positions (Zechner et al., 2012). In terms of energy metabolism, DG-containing acyl chains at sn-1 and sn-2 positions (1,2-DG) serves as an intermediate product for triglyceride (TG) synthesis. In terms of signal transduction, 1,2-DG is known to serve as an intracellular signaling molecule that activates several proteins including conventional and novel types of protein kinase C (PKC), Unc-13, RasGRP, and transient receptor potential channels (Kanoh et al., 1990; Nishizuka, 1992; Sakane et al., 2007; Goto et al., 2014). Consequently, DG acts as a hub between lipid metabolism and intracellular signaling.

**FIGURE 1:**
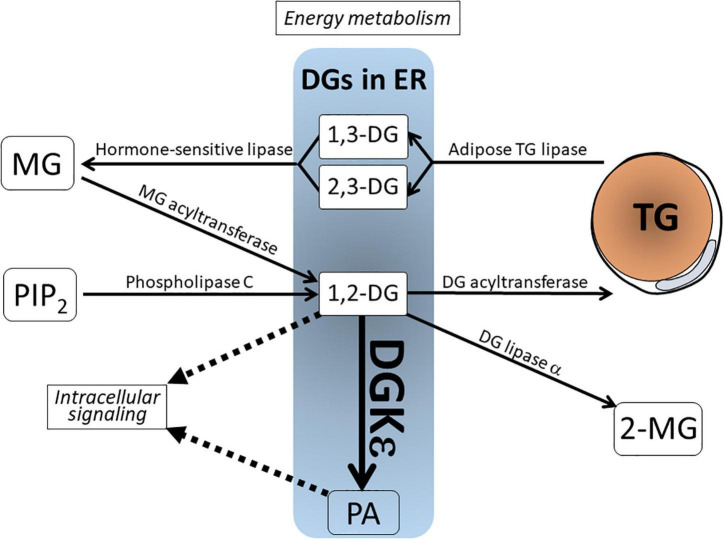
Schematic view of the DG in lipid metabolism and signal transduction. DGs in the endoplasmic reticulum (ER) include various species from distinct sources. 1,2-DG is derived from MG by MG acyltransferase and from phospholipase C-mediated PIP_2_ breakdown. 1,3-DG and 2,3-DG are lipolytic products of TG by adipose TG lipase. These DGs are not intermingled and separately metabolized. Note that 1,2-DG serves not only as an intermediate product for TG synthesis, but also as a lipidic second messenger. It is phosphorylated by the action of DGK to produce phosphatidic acid (PA). DG, diacylglycerol; DGKε, DG kinase ε; MG, monoacylglycerol; PA, phosphatidic acid; PIP_2_, phosphatidylinositol 4,5-bisphosphate; TG, triglyceride; 2-MG, 2-monoacylglycerol.

As depicted in Figure 1, DG derives from various sources. It is metabolized *via* several enzymatic pathways. In signal transduction cascade, 1,2-DG derives from phosphatidylinositol 4,5-bisphosphate (PIP_2_), a minor component of biological membrane, *via* phospholipase C (PLC) upon receptor stimulation (Goto et al., 2007; Blunsom and Cockcroft, 2020). In the course of energy metabolism, DG represents both a precursor of TG synthesis by DG acyltransferase (DGAT) and a product of TG hydrolysis by adipose triglyceride lipase (ATGL) (Zechner et al., 2012). In this regard, the former is 1,2-DG, whereas the latter is 1,3-DG or 2,3-DG. Therefore, these DGs are not intermingled and are separately metabolized. Other DG metabolizing enzymes include the following: (1) Hormone-sensitive lipase (HSL) hydrolyzes 1,3-/2,3-DG to monoacylglycerol (MG); (2) DG lipase α acts on 1,2-DG to produce 2-monoacylglycerol (2-MG); and (3) DG kinase (DGK) phosphorylates 1,2-DG to produce phosphatidic acid (PA). This catalysis is reversed by PA phosphatase, which dephosphorylates PA to generate DG (Goto et al., 2014; Sakane et al., 2018).

Of DG metabolizing enzymes, the significance of DGK has been reviewed comprehensively elsewhere (Sakane et al., 2007; Goto et al., 2014; Mérida et al., 2019). Briefly, DGK comprises an enzyme family composed of 10 isozymes in mammalian species. The DGK isozymes are classified into types I-V based on molecular structure. Each DGK isozyme shows a distinct enzymatic property, tissue distribution, and subcellular localization (Goto et al., 2007, 2014; Sakane et al., 2007; Topham and Epand, 2009). Considering these biochemical routes of DG metabolism described above, it is noteworthy that 1,2-DG, a substrate of DGK, lies at the crossroads of both TG precursor and second messenger. This arrangement implicates DGK in the regulation of both energy homeostasis and signal transduction.

Of the DGK family, DGKε is simplest in structure (64 kDa) and belongs to type III DGK (Tang et al., 1996). The most prominent feature of DGKε is its substrate specificity toward sn-2-arachidonoyl (20:4) DG species (Glomset, 1996; Tang et al., 1996; Nakano et al., 2016). Since polyphosphoinositides (PPIns) are composed mostly of sn-1-stearoyl-2-arachidonoyl acyl chains, arachidonoyl-DG is incorporated efficiently into PPIns (Glomset, 1996). However, catalysis of arachidonoyl-DG by DG lipase α generates 2-arachidonoyl glycerol (2-AG), an endocannabinoid that serves as endogenous “marijuana” in the brain (Bisogno et al., 2003; Tanimura et al., 2010). Therefore DGKε can be reasonably expected to participate in various pathophysiological events. This expectation is supported by several reports describing that DGKε is implicated in kidney diseases (Lemaire et al., 2013; Zhu et al., 2016), seizure (Rodriguez de Turco et al., 2001), inflammatory reaction (Yamamoto et al., 2014), and endoplasmic reticulum (ER) stress (Matsui et al., 2014).

From the perspective of subcellular localization, DGKε localizes to the ER (Kobayashi et al., 2007; Matsui et al., 2014; Nakano et al., 2016; Hozumi et al., 2017). Actually, ER is a central site for lipogenesis, from which lipid droplets protrude from the ER membrane stuffed with TG for storage. Therefore, ER-resident DGKε is thought to regulate 1,2-DG for signaling and energy metabolism. Recently, dynamic alterations of adipose tissue physiology under DGKε-deficient conditions have been reported (Nakano et al., 2018, 2020). This review specifically assesses the functional implications of DGKε in lipid orchestrated pathophysiology of adipose tissues under short-term and long-term high fat diet (HFD) feeding conditions.

## Diacylglycerol Kinase ε-Knockout Mice Show Obesity and Glucose Intolerance Under Short-Term High Fat Diet Feeding Conditions

To investigate metabolic syndrome, an abnormal lipid metabolism characterized by obesity and insulin resistance, HFD feeding is a useful model (Li et al., 2005; Lee et al., 2011; Benoit et al., 2013; Cantley et al., 2013). This model induces energy stress by overloading a fat-enriched diet, causing excessive TG accumulation in adipose tissues. In normal or healthy individuals, however, these obese conditions can be managed by homeostatic mechanism to keep the organism within a physiological range or at least to avoid immediate deterioration. In this regard, investigating how the phenotype is developed during the course of the model and identifying a primary event of a defect or mutation of a given gene is important because the final stage of metabolic syndrome shows a quite similar phenotype despite the distinct causes.

**FIGURE 2:**
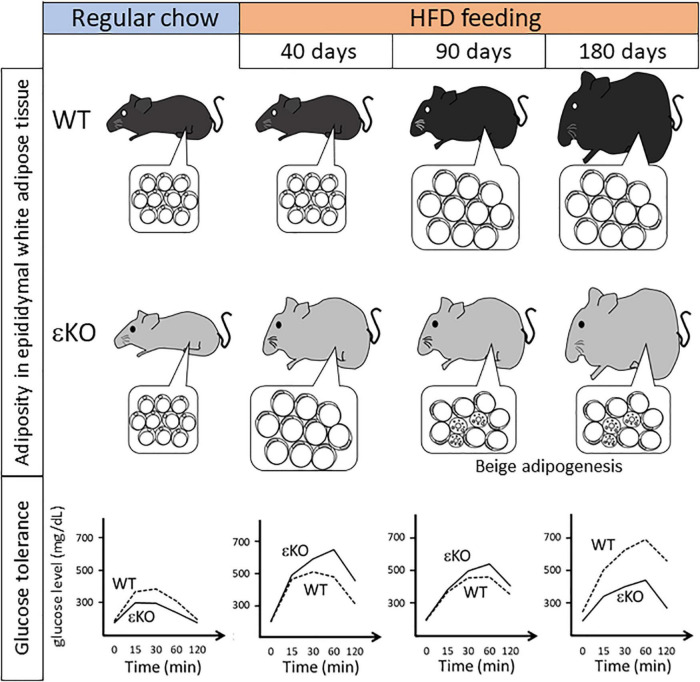
Summary of phenotypical alteration of DGKε-KO mice during the course of HFD feeding regimen. Pathophysiological alterations in body weight, adiposity in epididymal white adipose tissue (insets), and glucose tolerance are shown during the course of HFD feeding (Nakano et al., 2018, 2020). Note that in DGKε-KO mice under long-term HFD feeding beige adipogenesis as shown by multilocular cells is induced, which coincides with improved glucose tolerance. HFD, high fat diet; εKO, DGKε-KO mice; WT, wild-type mice.

Nakano et al. (2018) have examined how lipid metabolism is altered in DGKε-knockout (KO) mice under HFD feeding conditions. At 21 days of HFD feeding, a very early phase of obesogenic conditions, DGKε-KO mice tend to increase more body weight compared with wild-type (WT) mice. At 40 days of HFD feeding, DGKε-KO mice show considerable body weight gain and expanded mass of epididymal (i.e., visceral) white adipose tissue (WAT). At cellular level, the adipocyte cell size in DGKε-KO mice increases compared with that of WT controls. Consequently, WAT expansion is ascribed to accelerated lipid overloading in adipocytes of DGKε-KO mice. These results demonstrate that DGKε-KO mice are prone to obesity during early HFD feeding (Figure 2).

A clue to understanding this phenotype in DGKε-KO mice might be obtained based on results of glucose tolerance test (GTT) in Figure 2 (Nakano et al., 2018). Regular chow feeding leads to no difference in body weight gain between WT and DGKε-KO mice. It is particularly interesting that in GTT under regular chow feeding, plasma glucose kinetics reveals that glucose tolerance is enhanced in DGKε-KO mice compared with WT controls, although plasma insulin level tends to be lower in DGKε-KO mice. However, results in GTT after 40 days of HFD feeding show the inverse pattern: Glucose excursion is blunted in DGKε-KO mice compared with WT controls. The opposite patterns of glucose clearance in WT and DGKε-KO mice between regular chow and HFD feedings might be explained as follows: glucose uptake is intrinsically facilitated in DGKε-KO mice than WT controls, thereby leading to enhanced TG accumulation under energy excess conditions. This inference is supported by results demonstrating that oleic acid uptake is also facilitated in DGKε-deficient fat tissues (Nakano et al., 2018). Once TG storage exceeds the limit after 40 days of HFD feeding, obesity ensues, thereby causing insulin resistance, as described later.

Recent reports have described that TG overloading in adipocytes coincides with higher rates of basal lipolysis to dissipate extra energy (Wang et al., 2008; Kolditz and Langin, 2010). This machinery is crucially important for the maintenance of whole body energy homeostasis. The TG lipolysis in adipocytes is initiated by adipose TG lipase (ATGL), followed by hormone-sensitive lipase (HSL). ATGL catalyzes TG to generate DG, which is cleaved by HSL to release fatty acids. Intermediate product DG in TG lipolytic pathway represents 1,3/2,3-DG, which is distinct from and which is not intermingled with second messenger 1,2-DG (Zechner et al., 2012). However, in TG synthetic pathway, fatty acids are incorporated into MG to generate 1,2-DG, which is acylated immediately into TG by DGAT. This immediate conversion apparently attenuates 1,2-DG because excessive 1,2-DG might serve as a second messenger to induce aberrant activation of DG signaling pathway such as PKC. Actually, DGAT expression level is upregulated considerably in WT adipose tissue (Nakano et al., 2018). It is noteworthy that DGAT expression level is induced only slightly in DGKε-deficient adipocytes, which contrasts sharply to WT controls. Taken together, alterations of TG metabolizing enzymes in DGKε-KO mice under short-term HFD conditions can be summarized as follows: ATGL and HSL expression levels in TG lipolytic pathway are downregulated significantly, whereas the DGAT expression level in TG synthetic pathway is not induced. These alterations engender accumulation of TG and 1,2-DG.

How does TG and 1,2-DG accumulation exert effects on adipocytes? First, overaccumulation of TG in lipid droplets induces an inflammatory reaction in adipose tissues *via* TNF-α produced by infiltrated macrophages (Cinti et al., 2005; Lumeng et al., 2007). TNF-α facilitates Akt/PKB breakdown, thereby impairing insulin-dependent Akt/PKB signaling (Medina et al., 2005). Second, excessive accumulation of 1,2-DG triggers hyperactivation of DG-sensitive PKCθ in adipocytes, thereby increasing serine phosphorylation of insulin receptor substrate-1 (IRS-1). This increase exerts a negative effect on insulin signaling (Kim et al., 2004; Samuel and Shulman, 2012). Collectively, TG and 1,2-DG accumulation exerts “double negative effects” on insulin signaling, thereby inducing insulin resistance in DGKε-deficient adipose tissues under short-term HFD feeding. It must be described that other insulin-reactive organs such as liver and skeletal muscle of DGKε-KO mice exhibit no changes in PKC and Akt/PKB expression and activation status, suggesting that insulin signaling is specifically disturbed in adipose tissues of DGKε-KO mice under short-term HFD feeding conditions. This inference is consistent with results of an earlier study showing that skeletal muscle insulin sensitivity is unchanged in DGKε-KO mice (Mannerås-Holm et al., 2017).

## Beige Adipogenesis Is Induced in White Adipose Tissue of Diacylglycerol Kinase ε-Knockout Mice Under Long-Term High Fat Diet Feeding Conditions

Under short-term (40 days) HFD feeding, a presymptomatic phase of obesity in WT mice, DGKε-KO mice show severe obesity and insulin resistant phenotype, whereas WT mice remain normal. Earlier reports of some studies have described that obese phenotype occurs after 14 weeks of HFD feeding in WT animals (Benoit et al., 2013; Cantley et al., 2013). Next, Nakano et al. (2020) investigated the manner in which this phenotype is changed in DGKε-KO mice under long-term HFD feeding conditions (Figure 2). In WT mice, the obese phenotype becomes evident after 90 days of HFD feeding. The obesity worsens at 180 days. It is particularly interesting that the situation reverses in DGKε-KO mice: During the course of HFD feeding, plasma glucose kinetics of DGKε-KO mice in GTT exhibits the worst pattern at 40 days. At 90 days, however, it improves better and seems similar to that of WT controls. A surprising finding is that glucose tolerance in DGKε-KO mice is enhanced further at 180 days, and exhibits a much improved picture compared to that of WT controls: Plasma glucose kinetics in DGKε-KO mice at 180 days of HFD feeding is close to that observed in WT controls under regular chow feeding.

What sorts of changes are visible in WAT during HFD feeding? Epididymal WAT represents visceral WAT. It is useful to monitor changes in mass because it is demarcated clearly from surrounding tissues. In comparison, epididymal WAT mass shows no difference between WT and DGKε-KO mice under regular chow feeding. After 40 days of HFD feeding, however, the WAT mass in DGKε-KO mice increases approximately two-fold compared with that in WT mice. In sharp contrast to short-term (40 days) HFD feeding, it is noteworthy that under prolonged HFD feeding such as 90 and 180 days, the WAT mass in DGKε-KO mice decreases by nearly half in WT mice. Histological examination reveals that epididymal WAT is filled mainly with unilocular white adipocytes throughout the course of HFD feeding in WT mice. However, in DGKε-deficient WAT, UCP1-positive cells having multilocular vacuoles in abundant cytoplasm and round nuclei are scattered throughout the tissue at 90 and 180 days. Since UCP1 is a marker for beige/brown adipocyte (Harms and Seale, 2013), those findings suggest that beige adipogenesis or browning of white adipocytes is induced in DGKε-deficient WAT under long-term HFD feeding. Taking glucose tolerance data and histological findings together, a hypothesis can be proposed: In DGKε-KO mice under long-term HFD feeding, beige adipogenesis contributes to efficient energy dissipation, which enhances glucose tolerance. This hypothesis is supported by an earlier study showing that absence of functional beige adipocytes renders mice prone to obesity, insulin resistance, and hepatic steatosis upon HFD feeding (Cohen et al., 2014).

## Discussion

High fat diet studies reveal that under long-term (90 days ∼) HFD feeding conditions, beige adipogenesis is induced in white adipose tissue, which may contribute to enhanced glucose tolerance in DGKε-KO mice. Should DGKε be a therapeutic target for obesity? It is not so simple because DGKε-KO mice show severe obesity and insulin resistant phenotype under short-term (40 days) HFD feeding conditions. In addition, several questions remain unsolved, although regulatory roles of DGKε in adipose tissues have been elucidated gradually. First, glucose and fatty acid uptake is apparently facilitated in DGKε-KO mice on regular chow. How does DGKε regulate energy uptake? Second, protein expression of ATGL, a TG lipolytic enzyme, is downregulated under obese conditions. In this case, ATGL mRNA level remains unchanged in regular chow and HFD feedings (Nakano et al., 2018). Is ATGL protein degradation promoted in the absence of DGKε? Third, beige adipogenesis is induced in DGKε-deficient WAT under long-term HFD feeding. Is this beiging merely a homeostatic reaction against excess energy accumulation to dissipate extra energy? Or is it regulated directly by DGKε? Further studies must be conducted to address these points.

The primary function of beige/brown adipocytes is thermogenesis. These cells consume glucose and fatty acids to generate heat, instead of ATP production. Results of recent studies suggest that cold exposure facilitates thermogenesis by beige adipogenesis and browning of white adipocytes, in which UCP1 regulates uncoupled respiration to generate heat (Vegiopoulos et al., 2010; Bayindir et al., 2015). Examination of how beiging and browning are regulated by DGKε on exposure to cold is expected to be interesting.

## Author Contributions

TN performed the experiments in the original manuscript and summarized the results for the mini review. TN and KG constituted and wrote the manuscript. Both authors contributed to the article and approved the submitted version.

## Conflict of Interest

The authors declare that the research was conducted in the absence of any commercial or financial relationships that could be construed as a potential conflict of interest.

## Publisher’s Note

All claims expressed in this article are solely those of the authors and do not necessarily represent those of their affiliated organizations, or those of the publisher, the editors and the reviewers. Any product that may be evaluated in this article, or claim that may be made by its manufacturer, is not guaranteed or endorsed by the publisher.
